# Effect of Streptozotocin-Inducted Diabetes on the Pathophysiology of Enteric Neurons in the Small Intestine Based on the Porcine Diabetes Model

**DOI:** 10.3390/ijms21062047

**Published:** 2020-03-17

**Authors:** Michał Bulc, Jarosław Całka, Katarzyna Palus

**Affiliations:** Department of Clinical Physiology Faculty of Veterinary Medicine, University of Warmia and Mazury, Oczapowskiego Str. 13, 10-719 Olsztyn, Poland; jaroslaw.calka@uwm.edu.pl (J.C.); katarzyna.palus@uwm.edu.pl (K.P.)

**Keywords:** enteric nervous system, diabetes, neuropeptides, pig, gastrointestinal complications

## Abstract

Hyperglycemia is one of the main causes of diabetes complications. Gastrointestinal (GI) disturbances are one of the most frequent complications during diabetes. The porcine digestive tract possesses physiological and pathological similarities to the human digestive tract. This also applies to the innervation of the gastrointestinal tract. In this study, the influence of experimentally-inducted hyperglycemia was examined on the expression of vesicular acetylcholine transporter (VAChT), cocaine- and amphetamine-regulated transcript (CART), galanin (GAL), vasoactive intestinal polypeptide (VIP), and calcitonin gene-related peptide (CGRP) in the enteric nervous system (ENS) neurons in the small intestine of the pig. During the current study, an increased number of neurons containing CART, VIP, GAL, and CGRP under streptozotocin injection were observed. The augmentation of expression included all enteric plexuses present in the small intestine. The same results were obtained in the case of VAChT; namely, chronic hyperglycemia led to an increase in the number of neurons utilizing VAChT in all investigated plexuses. The obtained results suggested that the function of neuropeptides studied in this experiment depended on their localization in the ENS structures, as well as part of the GI tract. Diabetes led to alterations in the neurochemical phenotype of small intestine enteric neurons.

## 1. Introduction 

The gastrointestinal (GI) tract is innervated by both the central nervous system (CNS) and by the enteric nervous system (ENS), located within the wall of the digestive tract [1]. The ENS throughout the intramural plexuses (connected itself by a neuronal network) controls many digestive functions [1,2,3]. The ENS is characterized by high autonomy in the regulation of the GI tract function, but its functions may be regulated by CNS. The organization of the ENS clearly depends on the digestive tract region, but intraspecies differences have also been observed [4,5,6]. The intramural plexuses created by neuronal cell bodies are organized by two plexuses. In large animals, we can distinguish two plexuses in the stomach: myenteric plexus (MP) and submucosal plexus (SP), while in the gut (small and large), the latter is additionally divided into the inner submucosal plexus (ISP) and the outer submucosal plexus (OSP) [6,7]. The ENS in the small intestine contains several classes of neurons, including sensory neurons, interneurons, and motor neurons, through which smooth muscle activity, transmucosal fluid fluxes, local blood flow, nutrient resorption, and other functions are controlled and regulated. It should be stressed that the ENS controls gut functions independently from vago-vagal reflexes [8]. 

Furthermore, each neuron utilizes different neurotransmitters, which determine its chemical code [9]. Using immunohistochemical methods, numerous biologically active substances have been described in enteric neurons [10,11]. To date, more than 50 neurotransmitters and/or neuromodulators have been noted in the ENS [12]. One of the most widespread substances in the GI tract is acetylcholine (ACh) [12]. In order to identify neurons containing the ACh enzymes, synthesis or transport markers of ACh are used. Vesicular acetylcholine transporter (VAChT) is considered to be the main marker of cholinergic structures in the GI tract [13]. Its presence has been confirmed in neurons playing different functions, e.g., in excitatory motoneurons, interneurons, and sensory neurons [13]. Cocaine- and amphetamine-regulated transcript (CART) peptide, for the first time isolated from ovine hypothalamus [14], is extensively distributed in central and peripheral nervous systems, including the ENS [15,16]. Despite numerous studies, the accurate function of CART in GI physiology is poorly understood. Previous research revealed that CART could decrease gastric acid secretion and change colon motility [17]. Galanin (GAL) is an endogenous 29- or 30-amino-acid neuropeptide, with a broad expression on nervous system structures [18]. GAL has shown a broad spectrum of action in the digestive tract [19] by activating metabotropic G-coupled receptors, which changes permeability by opening membrane potassium ion channels. This results in the secretion of other neurotransmitters, as well as the regulation of gut motility and fluid circulation [20,21]. In turn, 28-amino acid-peptide vasoactive intestinal polypeptide (VIP) is one of the main inhibitory peptides in the GI tract [22]. VIP, through hyperpolarization, induces smooth muscle relaxation, decreases gastric acid secretion, and simultaneously increases bile secretion [23]. Moreover, VIP is a potent neuroprotective factor in the ENS [24]. In the proper function of gut physiology, the transduction of sensory and pain stimuli from the GI tract to the CNS is necessary. This type of information is transmitted via the afferent pathway, in which calcitonin gene-related peptide (CGRP) plays the main role [25]. Obviously, this is the main (but not the only) function that it performs by CGRP in the GI tract. Several studies have addressed numerous other important attributes of CGRP inside the digestive tract [26,27]. It should be mentioned that suppression of gastric acid secretion, hyperpolarization of the GI smooth muscular membrane, vasodilatation, and regulation of the absorption of nutrients from the gut are assigned to physiological functions of CGRP [28]. 

It is well-known that neurons located in both central and peripheral nervous systems exhibit a high degree of plasticity [29]. Many factors can damage neuronal tissue, and one of them is long-lasting high glucose levels. This, in turn, leads to injury of nerves (sensory, motors, and autonomic), which often occurs in the course of diabetes [30]. Due to the huge number of neurons creating the ENS, the digestive tract is often the victim of hyperglycemia [31]. Every organ of the GI tract can be damaged in the course of diabetes [31]. Symptoms, such as postprandial fullness, nausea, vomiting, bloating, early satiety, and abdominal pain, are typically described [32]. 

Studies on the pathomechanism of gastrointestinal complications in type 1 diabetes are often performed using animal models [33]. The well-known and often used rodent models of diabetes do not fully reflect the anatomical and physiological properties of the human digestive tract. Considering this fact, large mammals, including the pig, seem to be a better model to investigate gastrointestinal complications than small laboratory animals [34]. The pig is very useful in many aspects as a model for human physiology and pathophysiology because many organ systems of this species, as well as physiological and pathophysiological responses, resemble those observed in humans. Since diabetes occurs extremely rarely in pigs, exogenous injection of streptozotocin (STZ) is needed to induce diabetes [35]. After a few days of STZ injections, clear symptoms of diabetes are present [36,37,38,39,40,41]. In this experimental model, the main defining factor of diabetes is chronic hyperglycemia. Therefore, the goal of the present investigation was to induce diabetes in the pig model. Secondly, immunohistochemistry was used to assess changes in the number of enteric neurons in the small intestine expressing VAChT, CART, VIP, GAL, and CGRP immunoreactivity. Moreover, the role of peptides investigated in diabetes, especially in the GI tract, is poorly understood, and the data originate exclusively from rodents [31]. Taking the above data into consideration, it is reasonable to conduct this research using the pig diabetes model. In turn, the obtained results might be more representative in relation to humans. 

## 2. Results

### 2.1. General Condition

All pigs which received STZ developed diabetes within a few days, showing blood glucose concentration over 20 mmol/L. The mean glucose concentration in the diabetic group during the time of the experiment was 20.57 mmol/L ± 0.94, while, in the control group, it was at physiological level 5.07 mmol/L ± 0.12 (Table 1). It should be added that although glucose level in blood serum in experimental animals was remarkably higher than in controls, all pigs which received STZ survived the duration of the experiment in a good health, and none of the animals required exogenous insulin injections.

### 2.2. Cocaine- and Amphetamine-Regulated Transcript (CART) Distribution 

#### 2.2.1. Duodenum

**Figure 1:**
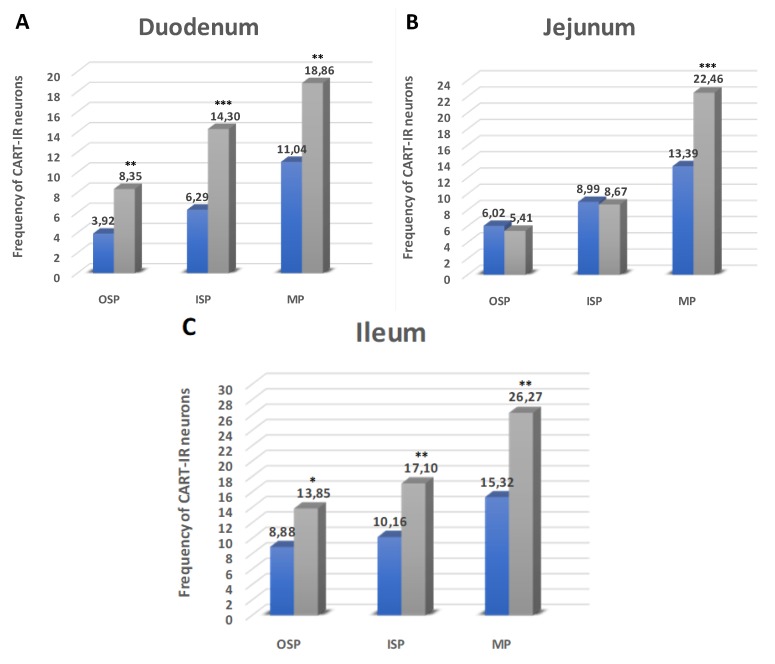
Diagram of the numbers of perikarya immunoreactive to Cocaine- and amphetamine-regulated transcript (CART) (**A–C**) of the control (blue bars) and experimental group (grey bars) in the particular parts of the small intestine. OSP – outer submucosal plexus, ISP—inner submucosal plexus, MP—myenteric plexus. * *p* < 0.05, ** *p* < 0.01, *** *p* < 0.001—indicate differences between all groups for the same neuronal populations.

**Figure 2:**
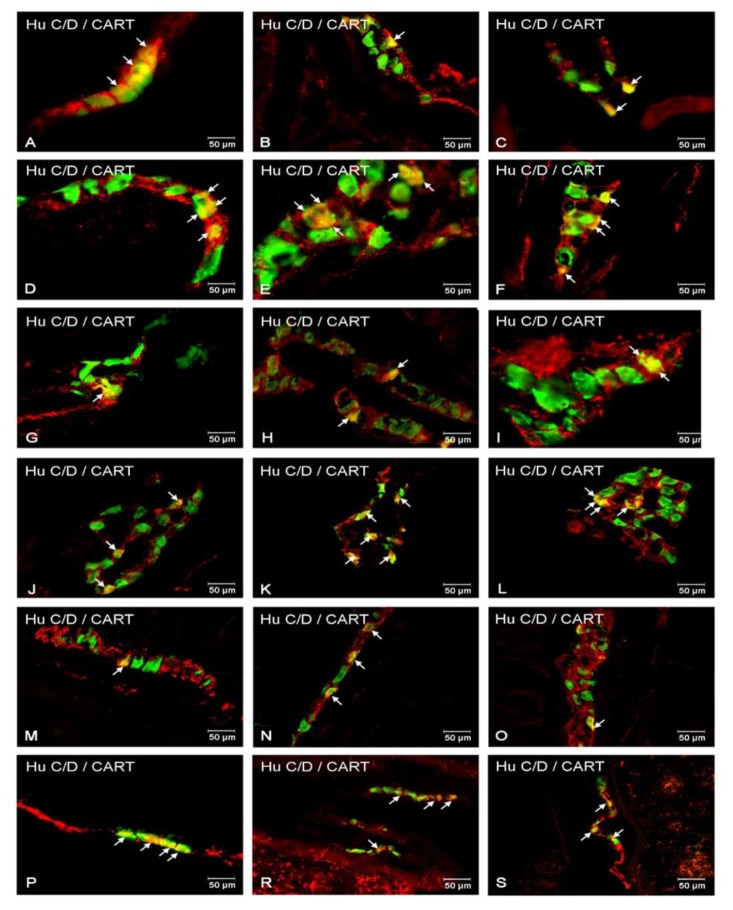
Immunofluorescent staining presenting CART immunoreactivity in cell bodies in the particular intramural plexuses in the small intestine in both control and experimental groups. All photographs have been created by the digital superimposition of two color channels; Hu C/D-positive—used here as a pan-neuronal marker (green), and CART-positive (red). The arrow shows perikaryon containing both examined substances. Myenteric plexus of the duodenum (**A,D**), jejunum (**B,E**), and ileum (**C,F**) under physiological condition (**A–C**) and after streptozotocin administration (**D–F**). Outer submucosal plexus of the duodenum (**G,J**), jejunum (**H,K**), and ileum (**I,L**) under physiological condition (**G–I**) and after streptozotocin administration (**J–L**). Inner submucosal plexus of the duodenum (**M,P**), jejunum (**N,R**), and ileum (**O,S**) under physiological condition (**M–O**) and after streptozotocin administration (**P–S**).

During the present investigation, CART occurred in both submucosal and myenteric plexuses in the duodenum (Figure 1A and Figure 2A,D,G,J,M,P). In the control group, neurons immunoreactive to CART (CART-IR) counted 11.04 ± 0.98% of neurons immunoreactive to Hu C/D in the myenteric plexus (MP) and increased in diabetic pigs to 18.86 ± 1.11% (Figure 1A and Figure 2A,D). In the submucosal plexuses, the total number of CART-IR neurons were slightly lower compared to MP. In the outer submucosal plexus (OSP) in control animals, the number of CART neurons was estimated at 3.92 ± 0.76% (Figure 1A and Figure 2G), while, in the experimental group, we observed a reduced level of CART-IR cell bodies (8.35 ± 1.56%) (Figure 1A and Figure 2J). Meanwhile, in the inner submucosal plexus (ISP), we observed a statistically significant increase in the experimental group compared to control ones (6.29 ± 0.56% vs. 14.30 ± 1.81%) (Figure 1A and Figure 2M,P).

#### 2.2.2. Jejunum

Also, in this part of the small intestine, CART-IR neurons were detected in all kinds of plexuses (Figure 1B and Figure 2B,E,H,K,N,R). In the MP, in control animals, neurons immunopositive to CART constituted 13.39 ± 1.09% (Figure 1B and Figure 2B) and increased in diabetes animals to 22.46 ± 2.46% (Figure 1B and Figure 2E). In turn, in the OSP, CART-IR neurons constituted 6.02 ± 0.78% of Hu C/D neurons, and their quantity did not change as a result of STZ injection (Figure 1B and Figure 2H,K). A similar situation was observed in the ISP, where, in the control group, the number of CART-IR cell bodies was estimated at 8.99 ± 0.87% and did not change in the experimental group (Figure 1B and Figure 2N,R).

#### 2.2.3. Ileum

In this part of the intestine, CART-IR neurons were also presented in all plexuses (Figure 1C and Figure 2C,F,I,L,O,S), and their total number was the highest of all small intestine segments. In the control animals inside the MP, CART-IR neurons were estimated at 15.32 ± 1.23% (Figure 1C and Figure 2C) and increased to 26.27 ± 2.64% in diabetic animals (Figure 1C and Figure 2F). Also, in the OSP, we noted an increased number of CART-IR neurons (8.88 ± 0.93% in control animals and 13.85 ± 1.56% in hyperglycemic animals) (Figure 1C and Figure 2O,L). In turn, in the ISP, we noted 10.16 ± 0.65% of CART-IR neurons in control animals (Figure 1C and Figure 2O) and 17.10 ± 2.07% in the experimental group (Figure 1C and Figure 2S).

### 2.3. Galanin (GAL) Distribution

#### 2.3.1. Duodenum

**Figure 3:**
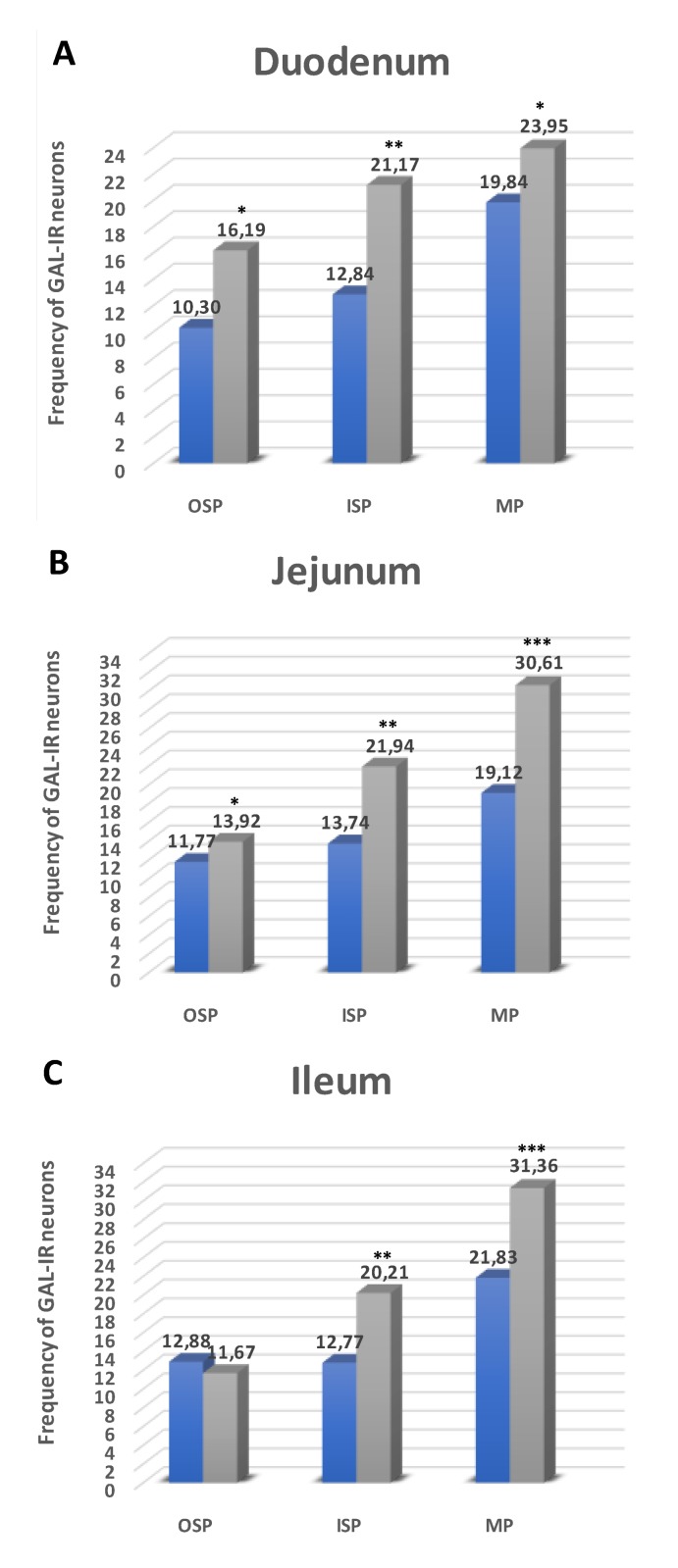
Schematic diagram of the numbers of perikarya immunoreactive to galanin (GAL) (**A–C**) of the control (blue bars) and experimental group (grey bars) in the particular parts of the small intestine. OSP – outer submucosal plexus, ISP—inner submucosal plexus, MP—myenteric plexus. * *p* < 0.05, ** *p* < 0.01, *** *p* < 0.001 – indicate differences between all groups for the same neuronal populations.

**Figure 4:**
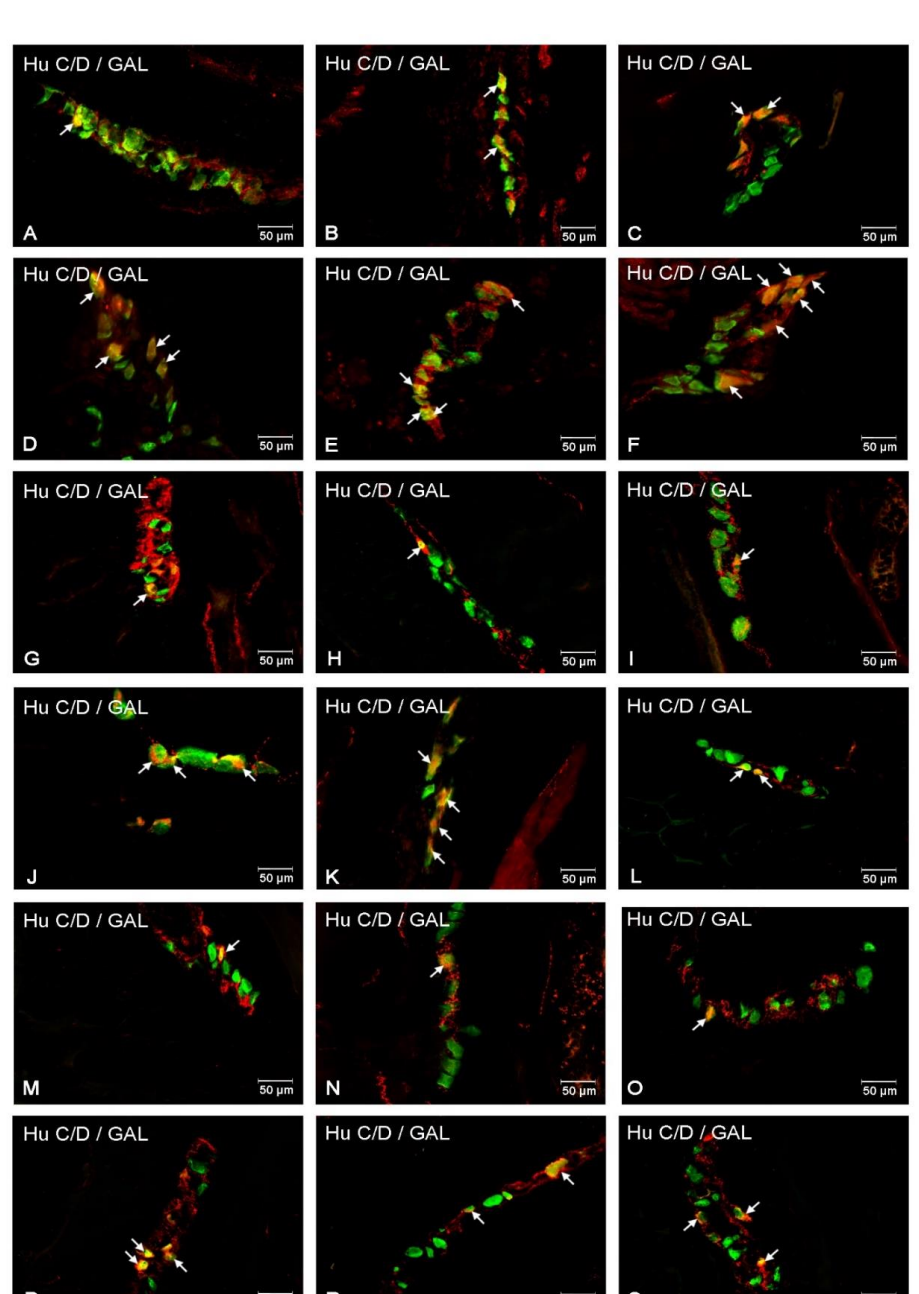
Immunofluorescent staining presenting GAL immunoreactivity in cell bodies in the particular intramural plexuses in the small intestine in both control and experimental groups. All photographs have been created by the digital superimposition of two color channels; Hu C/D-positive—used here as a pan-neuronal marker (green), and GAL-positive (red). The arrow shows perikaryon containing both examined substances. Myenteric plexus of the duodenum (**A,D**), jejunum (**B,E**), and ileum (**C,F**) under physiological condition (**A–C**) and after streptozotocin administration (**D–F**).Outer submucosal plexus of the duodenum (**G,J**), jejunum (**H,K**), and ileum (**I,L**) under physiological condition (**G–I**) and after streptozotocin administration (**J–L**). Inner submucosal plexus of the duodenum (**M,P**), jejunum (**N,R**), and ileum (**O,S**) under physiological condition (**M–O**) and after streptozotocin administration (**P–S**).

Another investigated substance was GAL. Neurons immunoreactive to GAL (GAL-IR) in control animals in the MP constituted 19.84 ± 0.78% of total Hu C/D positive neurons (Figure 3A and Figure 4A). Higher glucose blood concentration in experimental animals led to an increase in the number of GAL-IR neurons (to 23.95 ± 2.21%) (Figure 3A and Figure 4D). In the OSP, in healthy animals, the number of GAL-IR neurons was estimated at 10.30 ± 0.93% (Figure 3A and Figure 4G), while, in the experimental group, we noted an increased level of GAL-IR cell bodies (16.19 ± 1.57% (Figure 3A and Figure 4J). In turn, in the ISP, we observed statistically significant changes in the experimental group compared to control ones (12.84 ± 0.45% vs. 21.17 ± 1.13%) (Figure 3A and Figure 4M,P).

#### 2.3.2. Jejunum

In the jejunum, GAL-IR neurons were described in all enteric plexuses. Namely, in the MP, their number was estimated at 19.12 ± 0.67 in control (Figure 3B and Figure 4B) and 30.61 ± 2.87% in the experimental group (Figure 3B and Figure 4E). While, in the OSP, the total number of GAL-IR cell bodes amounted to 11.77 ± 0.94% in the control group (Figure 3B and Figure 4H) and increased slightly in the experimental group (13.92 ± 0.67%) (Figure 3A and Figure 4K). With regard to the ISP, the number of GAL-IR neurons was estimated at 13.74 ± 0.93% in control animals (Figure 3B and Figure 4N) and elevated in the diabetic group (21.94 ± 2.89%) (Figure 3B and Figure 4R).

#### 2.3.3. Ileum

The number of GAL-IR neurons in control animals inside the MP was estimated at 21.83 ± 2.06% (Figure 3C and Figure 4C), while, in hyperglycemic animals, their number was statistically higher (31.36 ± 2.27%) (Figure 3C and Figure 4F). In submucosal plexuses, we noted an increased number of GAL-IR neurons only in the ISP (12.77 ± 0.97% vs. 20.21 ± 2.37%) (Figure 3C and Figure 4I,L), while, in the OSP, the quality changes in GAL-IR neurons did not occur (Figure 3C and Figure 4O,S).

### 2.4. Vasoactive Intestinal Polypeptide (VIP) Distribution

#### 2.4.1. Duodenum

**Figure 5:**
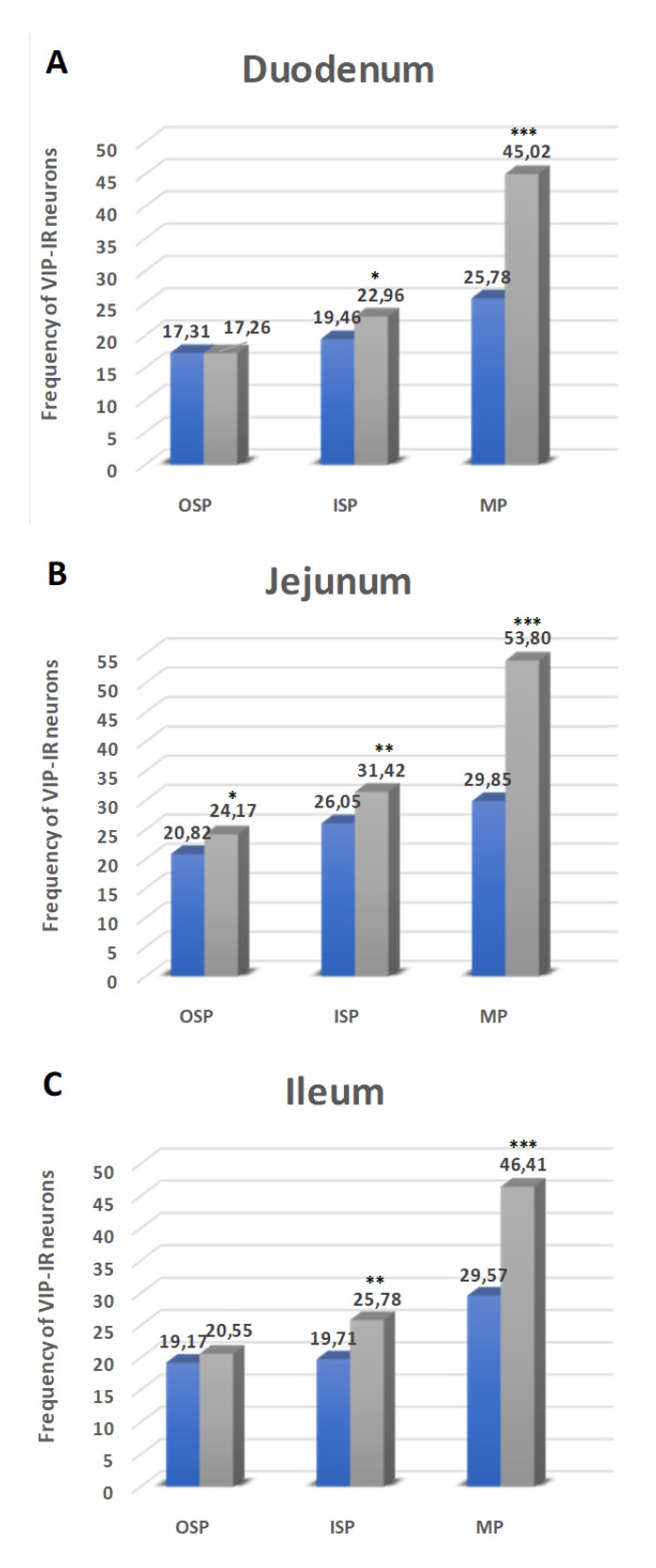
Schematic diagram of the numbers of perikarya immunoreactive to a vasoactive intestinal polypeptide (VIP) (**A–C**) of the control (blue bars) and experimental group (grey bars) in the particular parts of the small intestine. OSP – outer submucosal plexus, ISP—inner submucosal plexus, MP—myenteric plexus. * *p* < 0.05, ** *p* < 0.01, *** *p* < 0.001—indicate differences between all groups for the same neuronal populations.

**Figure 6:**
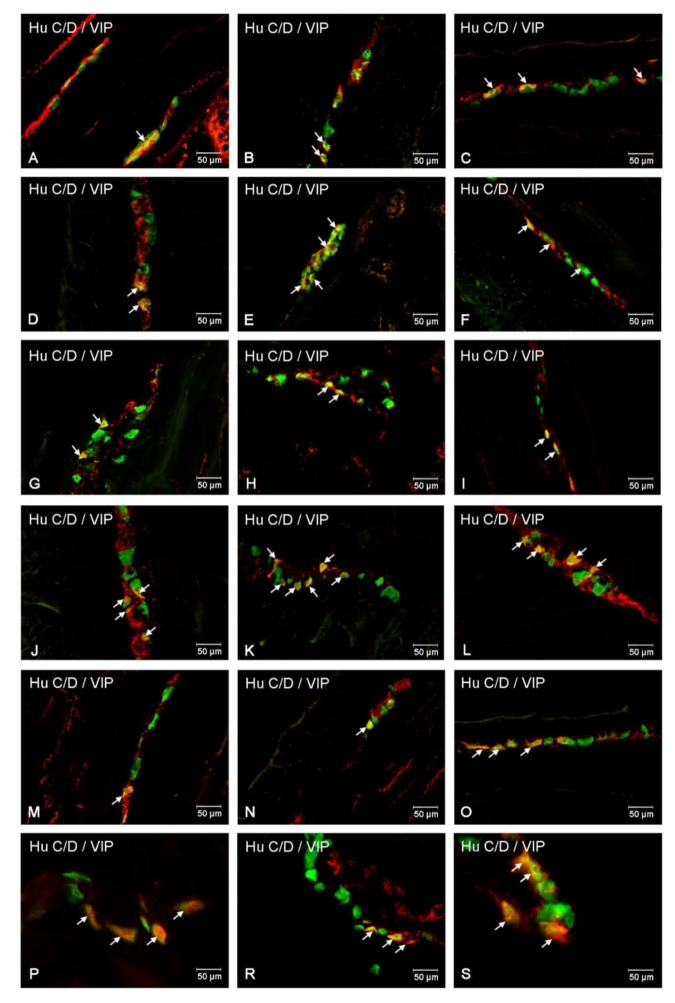
Immunofluorescent staining presenting VIP immunoreactivity in cell bodies in the particular intramural plexuses in the small intestine in both control and experimental groups. All photographs have been created by the digital superimposition of two color channels; Hu C/D-positive—used here as a pan-neuronal marker (green), and VIP-positive (red). The arrow shows perikaryon containing both examined substances. Myenteric plexus of the duodenum (**A,D**), jejunum (**B,E**), and ileum (**C,F**) under physiological condition (**A–C**) and after streptozotocin administration (**D–F**). Outer submucosal plexus of the duodenum (**G,J**), jejunum (**H,K**), and ileum (**I,L**) under physiological condition (**G–I**) and after streptozotocin administration (**J–L**). Inner submucosal plexus of the duodenum (**M,P**), jejunum (**N,R**), and ileum (**O,S**) under physiological condition (**M–O**) and after streptozotocin administration (**P–S**).

The next investigated substance was VIP, whose presence was described in all intestine plexuses. In the MP, in control animals, neurons immunoreactive to VIP (VIP-IR) constituted 25.78 ± 3.45% (Figure 5A and Figure 6A). Injection of STZ led to an increase in the number of VIP-IR neurons in the MP (45.02 ± 2.29%) (Figure 5A and Figure 6D). In submucosal plexuses, the increased population of VIP-IR neurons was visible only in the ISP (19.46 ± 1.56% vs. 22.96 ± 2.08%) (Figure 5A and Figure 6M,P). In the OSP, we had not observed statistically significant changes (Figure 5A and Figure 6G,J).

#### 2.4.2. Jejunum

VIP-IR neurons were the most numerous in the MP in both groups (29.85 ± 2.87% in control and 53.80 ± 3.32% in experimental animals) (Figure 5B and Figure 6B,E). In the OSP, the number of VIP-IR neurons was estimated at 20.82 ± 1.67% in the control group (Figure 5B and Figure 6H) and increased in experimental animals to 24.14 ± 1.64% (Figure 5B and Figure 6K). A similar increase was described in the ISP. Namely, in control animals, the population of VIP-IR neurons was estimated at 26.06 ± 2.09% and increased to 31.42 ± 2.98% in the experimental group, respectively (Figure 5B and Figure 6N,R).

#### 2.4.3. Ileum

In turn, in the ileum, VIP-IR neurons in the MP of control animals were estimated at 29.57 ± 1.56% (Figure 5C and Figure 6C) and increased in diabetic animals to 46.41 ± 2.92% (Figure 5C and Figure 6F). In the OSP, the number of VIP-IR neurons constituted 19.17 ± 1.64% in control (Figure 5C and Figure 6I) and did not show statistically significant changes in the diabetic group (20.55 ± 1.50%) (Figure 5C and Figure 6L). In turn, in the ISP, the number of VIP-IR neurons was estimated at 19.71 ± 1.29% in control animals (Figure 5C and Figure 6O) and increased to the level 25.78 ± 3.06% in the experimental group (Figure 5C and Figure 6S).

### 2.5. Calcitonin Gene-Related Peptide (CGRP) Distribution

#### 2.5.1. Duodenum

**Figure 7:**
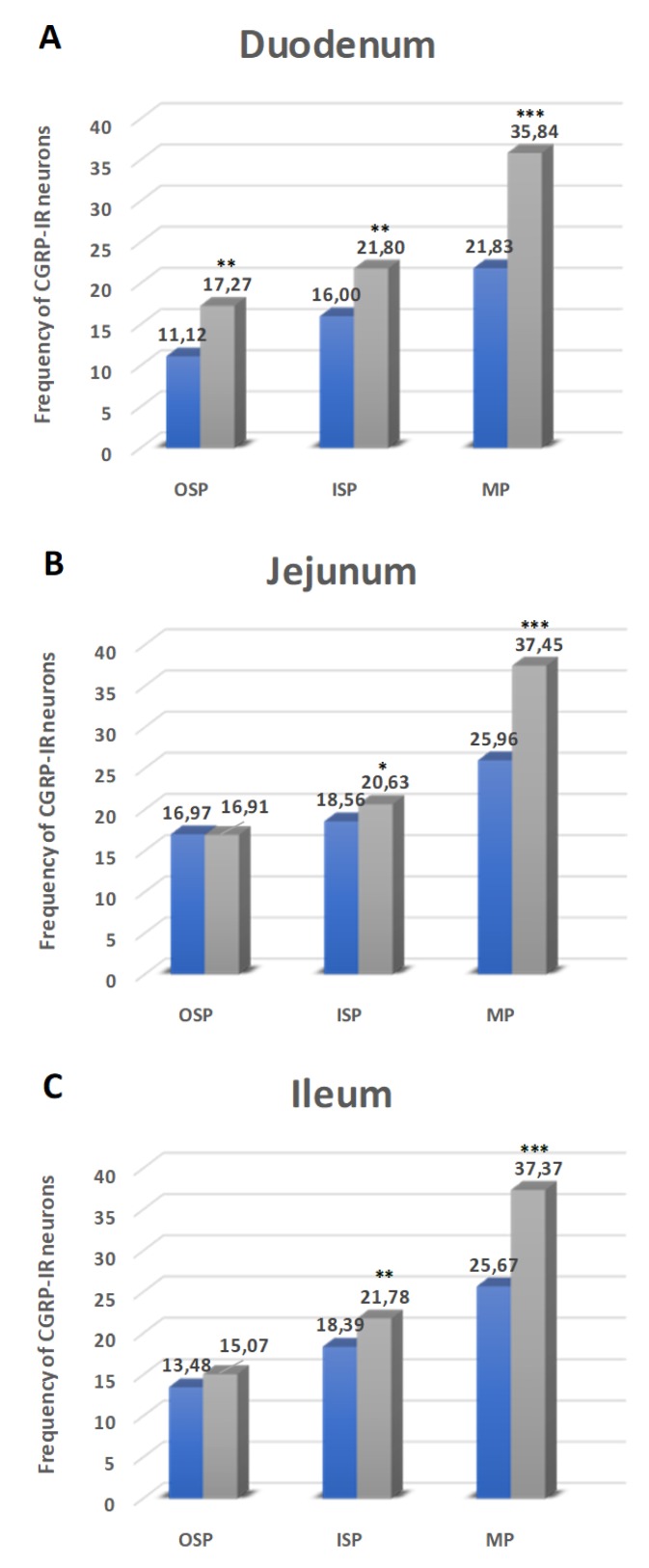
Schematic diagram of the numbers of perikarya immunoreactive to calcitonin gene-related peptide (CGRP) (**A–C**) of the control (blue bars) and experimental group (grey bars) in the particular parts of the small intestine. OSP – outer submucosal plexus, ISP – inner submucosal plexus, MP – myenteric plexus. * *p* < 0.05, ** *p* < 0.01, *** *p* < 0.001 – indicate differences between all groups for the same neuronal populations.

**Figure 8:**
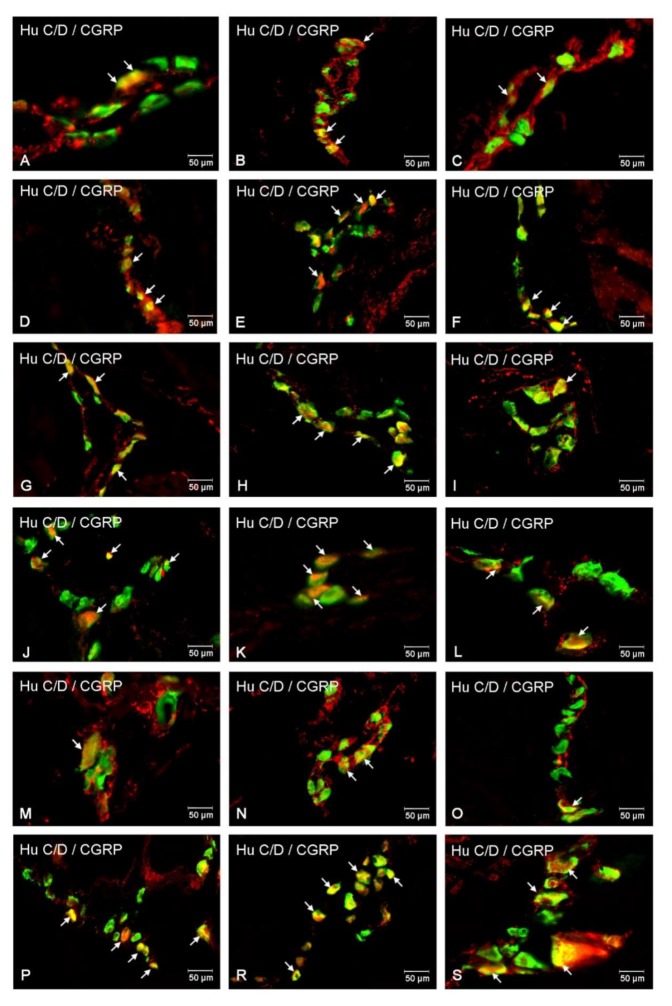
Immunofluorescent staining presenting CGRP immunoreactivity in cell bodies in the particular intramural plexuses in the small intestine in both control and experimental groups. All photographs have been created by the digital superimposition of two color channels; Hu C/D-positive—used here as a pan-neuronal marker (green), and CGRP-positive (red). The arrow shows perikaryon containing both examined substances. Myenteric plexus of the duodenum (**A,D**), jejunum (**B,E**), and ileum (**C,F**) under physiological condition (**A–C**) and after streptozotocin administration (**D–F**). Outer submucosal plexus of the duodenum (**G,J**), jejunum (**H,K**), and ileum (**I,L**) under physiological condition (**G-I**) and after streptozotocin administration (**J–L**). Inner submucosal plexus of the duodenum (**M,P**), jejunum (**N,R**), and ileum (**O,S**) under physiological condition (**M–O**) and after streptozotocin administration (**P–S**).

Another exanimated substance was CGRP. CGRP-immunoreactive neurons (CGRP-IR) in control animals inside MP were estimated at 21.83 ± 1.16% (Figure 7A and Figure 8A), while, in hyperglycemic animals, their number was statistically higher (35.84 ± 0.96%) (Figure 7A and Figure 8D). Also, in submucosal plexuses, we noted an increased number of CGRP-IR neurons. In the OSP, they constituted 11.12 ± 0.59% in control animals (Figure 7A and Figure 8G) and 17.27 ± 0.89% in experimental group (Figure 7A and Figure 8J). In turn, in the ISP, we noted 16.00 ± 0.66% of CGRP-IR neurons in control animals (Figure 7A and Figure 8M) and 21.80 ± 1.06% in the experimental group (Figure 7A and Figure 8P).

#### 2.5.2. Jejunum

In the MP, in control animals, CGRP-IR neuron constituted 25.96 ± 2.45% (Figure 7B and Figure 8B) and increased in diabetes animals to 37.45 ± 3.46% (Figure 7B and Figure 8E). In turn, in the OSP, in both groups, the number of CGRP-IR neurons was at a similar level (16.97 ± 0.99% vs. 16.91 ± 0.78%) (Figure 7B and Figure 8H,K). Regarding the ISP, the number of CGRP-IR cell bodies in the control group was estimated at 18.56 ± 0.80% (Figure 7B and Figure 8N), while, in experimental animals, the number of CGRP-IR neurons was higher (20.63 ± 1.27%) (Figure 7B and Figure 8R).

#### 2.5.3. Ileum

In this part of the intestine, CGRP-IR neurons were estimated at 25.67 ± 2.78% in the MP in control animals and increased at the level of 37.37 ± 2.90% in the experimental group (Figure 7C and Figure 8C,F). While in the OSP, we had not observed statistically significant changes between control and experimental groups (13.48 ± 0.98 vs. 15.07 ± 1.24%) (Figure 7C and Figure 8I,L). In turn, in the ISP, we noted 18.39± 0.65% of CGRP-IR neurons in control animals (Figure 7C and Figure 8O) and 21.78 ± 0.92% in the experimental group (Figure 7C and Figure 8S).

### 2.6. Vesicular Acetylcholine Transporter (VAChT)

#### 2.6.1. Duodenum

**Figure 9:**
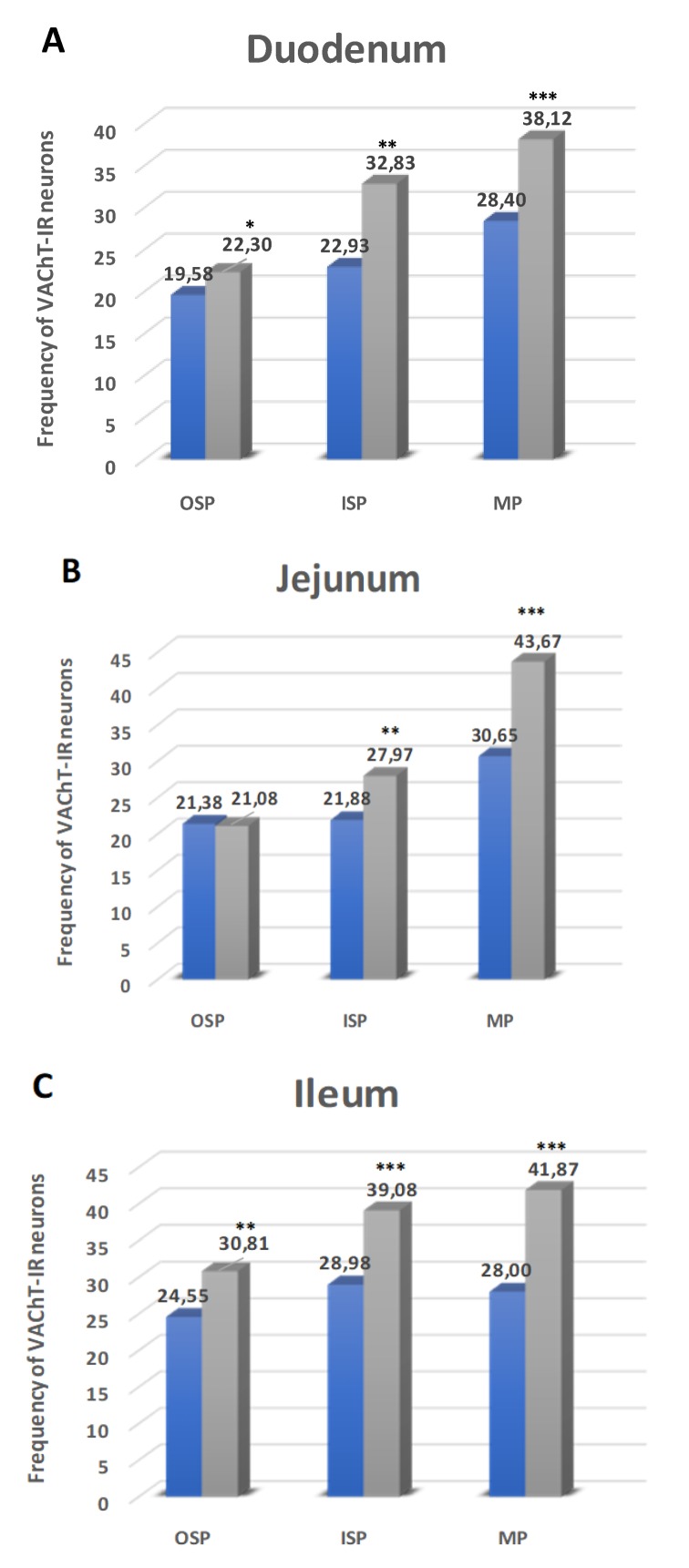
Schematic diagram of the numbers of perikarya immunoreactive to vesicular acetylcholine transporter (VAChT) (**A–C**) of the control (blue bars) and experimental group (grey bars) in the particular parts of the small intestine. OSP – outer submucosal plexus, ISP – inner submucosal plexus, MP – myenteric plexus. * *p* < 0.05, ** *p* < 0.01, *** *p* < 0.001 – indicate differences between all groups for the same neuronal populations.

**Figure 10:**
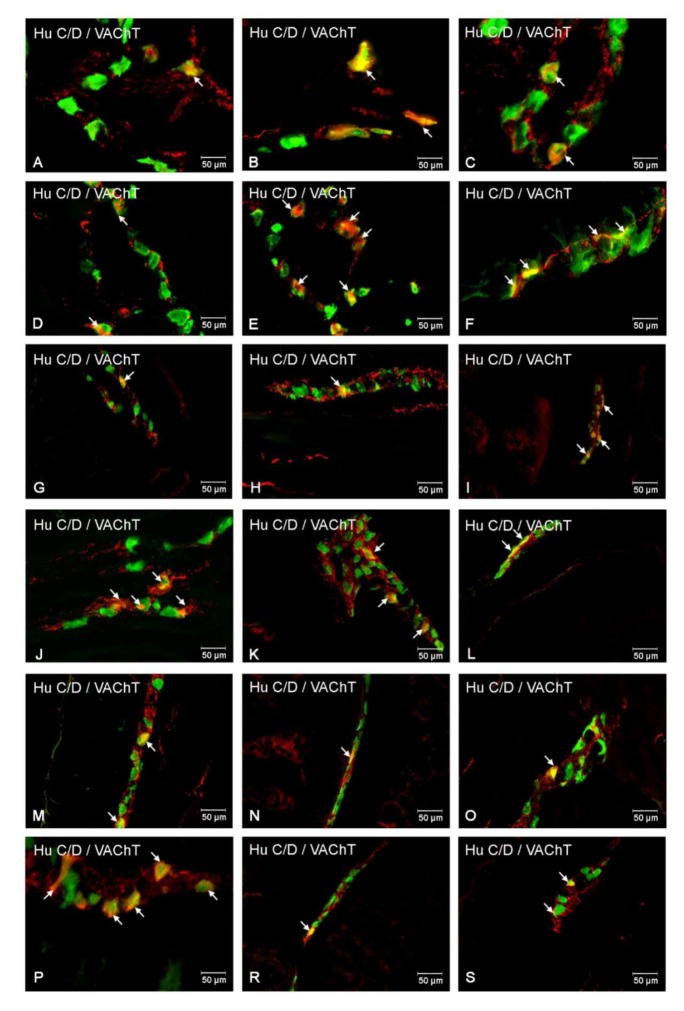
Immunofluorescent staining presenting VAChT immunoreactivity in cell bodies in the particular intramural plexuses in the small intestine in both control and experimental groups. All photographs have been created by the digital superimposition of two color channels; Hu C/D-positive—used here as a pan-neuronal marker (green), and VAChT-positive (red). The arrow shows perikaryon containing both examined substances. Myenteric plexus of the duodenum (**A,D**), jejunum (**B,E**), and ileum (**C,F**) under physiological condition (**A–C**) and after streptozotocin administration (**D–F**). Outer submucosal plexus of the duodenum (**G,J**), jejunum (**H,K**), and ileum (**I,L**) under physiological condition (**G-I**) and after streptozotocin administration (**J–L**). Inner submucosal plexus of the duodenum (**M,P**), jejunum (**N,R**), and ileum (**O,S**) under physiological condition (**M–O**) and after streptozotocin administration (**P–S**).

The last investigated substance was VAChT. In control group, VAChT-immunoreactive (VAChT-IR) neurons in the MP constituted 28.40 ± 0.94% (Figure 9A and Figure 10A) and increased in experimental pig to 38.12 ± 0.54% (Figure 9A and Figure 10D). With regard to the OSP, VAChT-IR neurons in the control group were estimated at 19.58 ± 1.87% (Figure 9A and Figure 10G) and increased to 20.30 ± 1.78% in hyperglycemic animals (Figure 9A and Figure 10J). In turn, in the ISP, we had also observed an increased number of VAChT-IR neurons (22.93 ± 2.98% in control and 32.83 ± 1.19% in experimental animals) (Figure 9A and Figure 10M,P).

#### 2.6.2. Jejunum

In the jejunum, the highest population and the highest increase of the number of VAChT-IR neurons were visible in the MP (30.65 ± 3.04% in control animals (Figure 9B and Figure 10B) and 43.67 ± 4.01% in the experimental group (Figure 9A and Figure 10E). In turn, in submucosal plexuses, only in the ISP, we observed a statistically significant increase of VAChT-containing neurons (21.88 ± 1.32% vs. 27.97 ± 1.56%) (Figure 9B and Figure 10N,R). While in the OSP, the changes were statistically insignificant (21.38 ± 1.24% vs. 21.08 ± 2.22%) (Figure 9B and Figure 10H,K).

#### 2.6.3. Ileum

In the ileum, we noted a statistically significant increase of VAChT-IR neurons in all investigated enteric plexuses. Namely, in the MP, in the control animals, the number of VAChT-IR neurons was estimated at 28.00 ± 2.97% and 41.87 ± 3.04% in experimental pigs (Figure 9C and Figure 10C,F). While, in the OSP, we observed 24.55 ± 1.45% of VAChT- positive neurons in the control group and 30.81 ± 2.03% in experimental animals (Figure 9C and Figure 10I,L). In turn, in the ISP, VAChT-IR neurons constituted 28.98 ± 1.95% in control pigs and 39.08 ± 2.49% in diabetic animals, respectively (Figure 9C and Figure 10O,S).

## 3. Discussion

Almost a hundred years ago, Canadian orthopedic surgeon Frederick G. Banting and his assistant Charles Best discovered insulin [41]. This ground-breaking discovery saved the lives of millions of people around the world. Unfortunately, the widespread use of insulin in diabetes therapy has not eliminated the development of diabetes complications [30,32]. The above-mentioned complications are a consequence of poor disease treatment, which leads to long episodes of elevated glucose levels [42]. Each organ and/or tissue can be damaged in the course of diabetes [42], particularly neural cells (both peripheral and central) may be destroyed during diabetes. The alimentary tract, due to its rich central innervation and a large number of neurons present in the wall, is often disabled due to increased glucose serum level. Diabetes gastroenteropathy has shown a wide spectrum of side effects (heartburn, diarrhea, fullness, delayed gastric emptying). Although these symptoms do not directly threaten life, they significantly decrease its quality. The exact mechanism of diabetes gastrointestinal complications is much less understood than even diabetic retinopathy or nephropathy [30,32,43].

The current study demonstrated the influence of chronically elevated glucose level, obtained as a result of streptozotocin injection, on the number of enteric neurons in the small intestine expressing selected neuropeptides in the pig as a model. This type of research is important because, as mentioned above, knowledge of the pathomechanism of diabetes damage is scarce, and neuropeptides might also be involved in these processes [33,44]. The most important question concerning diabetes research is the choice of an appropriate animal model. Obviously, diabetes research includes a broad spectrum of issues, starting with diabetes immunology [45], metabolism disorders [46,47], pharmacology treatment, insulin resistance through to diabetes complications [47]. The last problem predominantly develops as a result of high glucose level [47]. The available literature is predominated by experiments conducted with rodent models [33,44]. The availability of genetically modified mouse and rat models, which spontaneously develop diabetes, allows known molecular mechanisms of pancreatic insulin-producing cells to be destroyed, especially with reference to type I diabetes. In these cases, a rodent model is an irreplaceable tool for a better understanding of diabetes immunology [48]. To examine the side effects of hyperglycemia, pancreatic β cells can be destroyed. Chemically-induced elevated blood glucose concentration seems to be a safe and effective method of diabetes induction. It should be added that this way of obtaining hyperglycemia is commonly used in rats and mice [49], although the current study used the pig as a model to research diabetes gastrointestinal complications. It should be emphasized that swine are becoming increasingly more often used as models in the biomedical study [34]. In the current study, we achieved the intended goal. Firstly, streptozotocin was effective in hyperglycemia induction in pigs. Secondly, besides an increased glucose level, we did not observe abnormalities in the general health condition. However, it is very important that the obtained results could be applied to humans. Without a doubt, the alimentary tract in pigs in terms of anatomical and histological structure, as well as physiological processes, is more suitable to this type of research than the rodent GI tract. Likewise, the length of the GI, particularly the small intestine in the pig, resembles human GI properties. An important feature is also the fact that the pig, like humans, is an omnivorous animal. Taking everything into consideration, the choice of the animal model in the current study was reasonable and appropriate.

The appropriate function of the gastrointestinal tract is conducted by bilateral regulation between both central and enteric nervous systems [1,5]. In turn, communications between each neuron are fulfilled by a broad spectrum of neurotransmitters [9]. Moreover, neurotransmitters are also responsible for sending information between neurons. One of the most important functions executed by neuronal cells through synthesis and release of neurotransmitters is providing normal neuron functions under unfavorable conditions during pathological processes [29,50]. Taking this into consideration, the current work focused on changes in the number of enteric neurons synthesizing selected neurotransmitters in response to hyperglycemia. Additionally, the domestic pig was used as an experimental animal model for the first time. All examined biologically active substances were detected in the investigated area of the GI tract. Naturally, the number of neurons immunoreactive to a particular neurotransmitter was different, both in each segment of the GI tract as well as in individual plexuses. The first substance assayed was CART. This anorectic peptide is widely expressed in nervous tissue and outside the nervous system [15,16,17]. In the current study, its expression was confirmed inside enteric plexuses. As a response to hyperglycemia, an increased number of neurons expressing CART were observed in particular neurons within the myenteric plexuses. Previous studies also showed that CART was involved in the control of many pathological conditions within the GI tract. For instance, axotomy [51] (an overdose of acrylamide) has significantly enhanced the expression of CART in ENS structures in the alimentary tract [50]. A characteristic feature of the above pathologies is the presence of inflammatory conditions. In addition, diabetes, through chronically elevated glucose levels, has led to the activation of the immune system [45]. It is not clear if CART plays a pro/or anti-inflammatory role, but this peptide is certainly involved in pathological changes in the gut during diabetes.

During the present study, an increased number of VIP-containing neurons was noted in almost all investigated areas. Notably, myenteric plexuses were places in which the augmentation of the number of VIP-IR neurons was the most visible. Previous studies in diabetic rats, which were focused on the ileum, are similar to the current data. Namely, in rats, an increase in VIP immunoreactivity also takes place [52]. Generally, the distribution and number of VIP-IR neurons depend on the fragment of the GI tract and animal species [53]. However, the function of VIP in the alimentary tract is less variable. The most important role seems to be an inhibitory action [54]. This inhibitory action includes smooth muscle relaxation and a decrease in gastric acid and intestinal fluid secretion [51]. Certain functions of VIP are revealed only during pathological states. In the previous paragraph, inflammation during diabetes was discussed. In this context, VIP might also be considered an anti-inflammatory agent. Inflammation is usually associated with increased cytokine synthesis [55]. Previous studies have shown that VIP is able to down-regulate this process through a decrease in macrophage activity [56]. Moreover, VIP may be responsible for changes in blood permeability, which is important in the development of gastric complications [53]. An increased number of VIP-positive neurons can be linked with the neuroprotective action of this peptide. Hyperglycemia often leads to severe neuron damage, including enteric neurons [30]. In turn, VIP, through activation of glial cells and stimulatory effect on anti-inflammatory mediator secretion, protects neurons and enables them to survive in adverse environmental conditions [56]. In neuroprotection and anti-inflammatory processes, another investigated peptide is also involved, mainly GAL [19]. Following diabetes induction, a significant increase in GAL expression in all parts of the small intestine was observed. In rats, similar changes have been observed in the ileum [57] and colon [58]. Moreover, GAL is also able to modulate the secretion of other neurotransmitters, including VIP, and affect the motor activity of the intestines [20,21].

One of the more troublesome digestive tract complaints occurring as a complication of diabetes is abdominal pain [5,59]. Nociception information is sent from the GI tract to the brain via a multistep pathway. In the transmission of pain signals, different molecules are involved [60]. One of them is CGRP, which is considered as a marker of primary afferent neurons in the alimentary tract and plays a crucial role in the regulation of these processes. In the current study, an increased number of neurons coding CGRP was observed. Alterations in the expression of CGRP have also been described in the gut of diabetic rats [61,62]. Interestingly, in rodents, CGRP in the ileum has decreased following diabetes [63]. This discrepancy may be caused by a different threshold of pain stimuli in rodents and large animals. Moreover, it cannot be assumed that this situation is permanent and immutable. It is well-established that insulin supplementation can reverse neurochemical coding inside neurons [64]. The acute or/and chronic episodes of hyperglycemia, during which toxic glucose action is revealed, translates into appropriate changes in the number of neurons containing adequate biologically active substances.

The final substance examined in the study was VAChT. Acetylcholine is one of the most commonly distributed neurotransmitters in the GI tract. It is found in different classes of enteric neurons, including excitatory motoneurons, interneurons, and, finally, in sensory neurons [65]. In the current study, VAChT was used as a marker of cholinergic neurons. It was dictated by the fact that VAChT-positive neurons have been confirmed in the pig GI tract, and their number was higher than neurons immunoreactive to another cholinergic marker, acetylcholine transferase (ChAT) [66]. The present study showed that after six weeks, hyperglycemia led to an increase in the number of VAChT-containing neurons. These results were in agreement with previous studies in which the density of cholinergic innervation was reported to increase in diabetes in the jejunum, ileum (myenteric plexus), and muscularis propria of the duodenum in rats and non-obese mice [67,68,69]. Since acetylcholine is a strong factor acting on muscle contraction, it is highly probable that an increase in the number of VAChT-positive structures (especially in the myenteric plexuses) may provoke disturbances of contractility observed in diabetes.

The present study revealed changes in the number of enteric neurons immunoreactive to selected substances occurring in the small intestine following streptozotocin-induced diabetes in pigs. The results, at least, partially corresponded with clinical symptoms of GI-tract disturbances in diabetes. The increase in VAChT might be responsible for improper motor activity in the gut, which results in disorders in the resorption and movement of digestive content. While increasing VIP and GAL immunoreactivity, molecules showed neuroprotective and pro-inflammatory properties, confirming that hyperglycemia leads to inflammatory conditions and damages autonomic neuronal cells. In turn, CGRP probably induced current in sensory endings and potentiates pain stimuli. Moreover, the current study confirmed the utility of the pig as a model for research into metabolic diseases. To date, the type 1 STZ-induced hyperglycemic/diabetic swine model has been mostly validated in the research of the cardiovascular complications of the disease [40], while neuronal complications in this model are not fully elucidated. The use of pigs as a model for researching diabetic complications also results from many similarities regarding anatomical and physiological aspects. Namely, the number of beta cells in the pancreas of pigs is very similar to the amount observed in humans; also, the blood supply in this organ is very similar between the pig and human. Such similarities do not occur in rodent pancreas [34,44]. Moreover, in pigs, glucose metabolism and insulin secretion also remain at a very similar level as in human diabetic patients [34]. Taking into account the above data and the fact that the pig, like a human, is an omnivore animal, we can assume that the changes in the ENS that we observed in this study may be related to humans.

## 4. Materials and Methods

Ten juvenile female pigs of the White Large Polish breed, weighing from 17 to 20 kg, were used in the experiment. After acclimatization directly before diabetes induction, the animals were divided into two groups: the diabetic group (D, *n* = 5) and the control group (C, *n* = 5). The treatment of animals was conducted in compliance with the instructions of the Local Ethical Committee in Olsztyn (Poland) (decision number 13/2015/DTN, 30. 10. 2015) and according to the Act for the Protection of Animals for Scientific or Educational Purposes of 15 January 2015 (Official Gazette 2015, No. 266), applicable in the Republic of Poland with special attention paid to minimizing any stress reaction.

After a week, diabetes was induced, as previously described [35,36,37]. Streptozotocin (STZ) (Sigma-Aldrich, St Louis, MO, USA, S0130), 150 mg/kg of body weight, was dissolved in a freshly prepared disodium citrate buffer solution (pH = 4.23, 1 g streptozotocin/10 mL solution). Animals were anesthetized, and the solution was administrated via an intravenous needle inserted into an ear with continuous infusion for approximately 5 min. To avoid gastrointestinal complications (mainly nausea and vomiting after streptozotocin injection), animals fasted for 18 h before the experiment. The control pigs were injected with equal amounts of the vehicle (citrate buffer). In order to avoid severe episodes of hyperglycemia, 250 mL of 50% glucose solution per animal was administered. After inducing diabetes, the animals were kept under standard laboratory conditions. They were fed standard fodder and had free access to water. The blood glucose concentration was estimated using an Accent-200 (Berlin, Germany) biochemical analyzer, with the colorimetric measurement at a wavelength of 510 nm/670 nm. For this aim, capillary blood from the ear was collected. The plasma glucose level was measured prior to the experiment initiation in both control and experimental groups. The next measurement was made 48 h after the injection of streptozotocin. Subsequent measurements of glucose levels were measured weekly until the end of the experiment.

Six weeks after streptozotocin injection, pigs were anesthetized via intravenous administration of pentobarbital (Vetbutal, Biowet, Poland) and perfused transcardially via the ascending aorta with freshly prepared 4% paraformaldehyde in 0.1 M (molar) phosphate buffer (pH 7.4). After perfusion, the small intestines were removed. Approximately, 2 cm fragments of duodenum, jejunum, and ileum were post-fixed by immersion in the same fixative for 10 min, then washed with 0.1 M PB (pH 7.4) over 2 days and finally transferred and stored at 400°C in an 18% buffered sucrose solution (pH 7.4), containing 0.001 % natrium azide. The tissues were then kept at -800°C until further processing. Frozen samples were cut in a cryostat (Microm HM 560 cryostat (Carl Zeiss, Germany) into 12-µm-thick sections and mounted on chrome alum-coated slides. Sections were processed by applying the routine double immunofluorescence technique. After drying at 32°C for 45 min, the sections were rinsed in a phosphate buffer containing 0.8% sodium chloride and 0.02% potassium chloride (PBS, 3 × 10 min.) and incubated in 10% normal goat serum in PBS with 0.3% Triton X-100 (Sigma, St. Louis, MO, USA) and 1% bovine serum albumin (BSA; Sigma, St. Louis, MO USA) for 20 min. The sections were then incubated overnight at 4°C with primary antibodies diluted in PBS containing 0.3% Triton X-100 and 1% BSA raised against Hu C/D (mouse polyclonal, Invitrogen, Waltham, MA, USA; Cat # A-212711:1.000; working dilution 1: 1000) and/or GAL (rabbit polyclonal, Merck Millipore, Billerica, MA USA; Cat. # AB 2233; working dilution 1:2000), VIP (rabbit polyclonal, Biomol, Hamburg, Germany; Cat # VA1285; working dilution 1:5000), CGRP (rabbit polyclonal, Merck Millipore, Billerica, MA, USA; Cat. # AB15360; working dilution 1:4000), CART (rabbit polyclonal, Phoenix Pharmaceuticals, Inc., Burlingame, Raleigh, NC, USA; Cat. # H-003-61; working dilution 1: 8000), VAChT (rabbit polyclonal, Phoenix Pharmaceuticals, Inc., Burlingame, Raleigh, NC, USA Cat. # H-V006; working dilution 1:2000). On the following day, the sections were rinsed (PBS, 5 × 15 min) and incubated with secondary antibodies (in PBS containing 0.25% BSA and 0.1% Triton X-100) for 4 h (Alexa Fluor 488 nm donkey anti-mouse, Invitrogen, Waltham, MA, USA; Cat # A21202; working dilatation; 1:1000 and Alexa Fluor 546 nm goat anti-rabbit, Invitrogen, Waltham, MA,USA; Cat # A11010; working dilution 1:1000). Next, the tissues were rinsed (PBS, 3 × 5 min) and covered with a polyethylene glycol/glycerine solution containing DABCO (Sigma, St. Louis, MO, USA). Standard controls, i.e., pre-absorption for the neuropeptide antisera (20 μg of appropriate antigen per 1 mL of the corresponding antibody at working dilution), as well as omission and replacement of the respective primary antiserum with the corresponding non-immune serum, completely abolished immunofluorescence and eliminated specific staining.

Immunostained neurons were analyzed using an Olympus BX51 microscope equipped with epi-illumination fluorescence filters. Photographs were taken by a digital monochromatic camera (Olympus XM 10). The microscope was equipped with the cellSens Dimension Image Processing software (Olympus, Hamburg, Germany). For determination of the percentage of VIP, CART, GAL, CGRP, and VAChT-LI neurons, at least 500 perikarya with a clearly visible nucleus immunoreactive to Hu C/D in the enteric plexuses from each animal were investigated. The obtained results were pooled and presented as mean ± SEM. To avoid double-counting of the same perikarya, the investigated sections of the intestine were located at least 100 μm apart. The data pooled from all animal groups were statistically analyzed using Statistica 13 software (StatSoft Inc., Tulsa, OK, USA) and expressed as a mean ± standard error (SEM) of the mean. Significant differences were evaluated using Student’s t-test for independent samples (* *p* < 0.05, ** *p* < 0.01, and *** *p* > 0.001).

## 5. Conclusions

We concluded that streptozotocin in a single dose (150 mg/kg) was able to induce diabetes in pig and suggested that STZ-injected pigs might serve as an essential model in early studies of pathological changes under hyperglycemic conditions in the peripheral nerve system. In our study, we supplied immunohistochemical evidence that six weeks of permanent hyperglycemia led to serve changes in the chemical phenotyping of enteric neurons in the small intestine of a pig. The increase of investigated peptides might be pivotal in the development of gastrointestinal complications. This finding provided the background for more detailed research on neuropeptides participation in the development of pathogenesis of intestinal changes, as well as reference data for further, pre-clinical studies on hyperglycemia-related gastrointestinal disturbances in the species more closely related to humans. Obviously, more detailed studies are needed to elucidate the precise mechanism and function playing by neuropeptides in GI complication development.

## Figures and Tables

**Table 1:** Serum glucose levels after induction of diabetes and glucose concentration after streptozotocin administration (from 1 week to 6 weeks).

Date	Control Group mg/dL	SEM ±	Experimental Group mg/dL	SEM ±
Before streptozotocin injection	90.18	0.10	90.4	0.10
1 week after streptozotocin injection	91.44	0.10	312.48	0.38
2 weeks after streptozotocin injection	88.38	0.18	372.96	0.24
3 weeks after streptozotocin injection	93.42	0.06	388.44	0.27
4 weeks after streptozotocin injection	95.58	0.12	361.44	0.09
5 weeks after streptozotocin injection	87.12	0.32	400.68	1.21
6 weeks after streptozotocin injection	93.6	0.1	386.1	1.11

## References

[1] Furness J.B., Callaghan B.P., Rivera L.R., Cho H.J. (2014). The enteric nervous system and gastrointestinal innervation: Integrated local and central control. Adv. Exp. Med. Biol..

[2] Furness J.B. (2012). The enteric nervous system and neurogastroenterology. Nat. Rev. Gastroenterol. Hepatol..

[3] Furness J.B. (2008). The enteric nervous system: Normal functions and enteric neuropathies. Neurogastroenterol. Motil..

[4] Schemann M., Neunlist M. (2004). The human enteric nervous system. Neurogastroenterol. Motil..

[5] Furness J.B. (2006). The organisation of the autonomic nervous system: Peripheral connections. Auton Neurosci..

[6] Arciszewski M.B., Barabasz S., Skobowiat C., Maksymowicz W., Majewski M. (2009). Immunodetection of cocaine- and amphetamine-regulated transcript in the rumen, reticulum, omasum and abomasum of the sheep. Anat. Histol. Embryol..

[7] Zacharko-Siembida A., Arciszewski M.B. (2014). Co-expression patterns of cocaine- and amphetamine-regulated transcript (CART) with neuropeptides in dorsal root ganglia of the pig. Anat. Histol. Embryol..

[8] Furness J.B. (2003). Intestinofugal neurons and sympathetic reflexes that bypass the central nervous system. J. Comp. Neurol..

[9] Lomax A.E., Furness J.B. (2000). Neurochemical classification of enteric neurons in the guinea-pig distal colon. Cell Tissue Res..

[10] Clerc N., Furness J.B., Li Z.S., Bornstein J.C., Kunze W.A. (1998). Morphological and immunohistochemical identification of neurons and their targets in the guinea-pig duodenum. Neuroscience.

[11] Furness J.B. (2000). Types of neurons in the enteric nervous system. J. Auton Nerv. Syst..

[12] Lomax A.E., Zhang J.Y., Furness J.B. (2000). Origins of cholinergic inputs to the cell bodies of intestinofugal neurons in the guinea pig distal colon. J. Comp. Neurol..

[13] Li Z.S., Furness J.B. (1998). Immunohistochemical localisation of cholinergic markers in putative intrinsic primary afferent neurons of the guinea-pig small intestine. Cell Tissue Res..

[14] Kristensen P., Judge M.E., Thim L., Ribel U., Christjansen K.N., Wulff B.S., Clausen J.T., Jensen P.B., Madsen O.D., Vrang N. (1998). Hypothalamic CART is a new anorectic peptide regulated by leptin. Nature.

[15] Wierup N., Gunnarsdóttir A., Ekblad E., Sundler F. (2007). Characterisation of CART-containing neurons and cells in the porcine pancreas, gastro-intestinal tract, adrenal and thyroid glands. BMC Neurosci..

[16] Ekblad E. (2006). CART in the enteric nervous system. Peptides.

[17] Ekblad E., Kuhar M., Wierup N., Sundler F. (2003). Cocaine- and amphetamine-regulated transcript: Distribution and function in rat gastrointestinal tract. Neurogastroenterol. Motil..

[18] Hökfelt T., Tatemoto K. (2008). Galanin 25 years with a multitalented neuropeptide. Cell. Mol. Life Sci..

[19] Arciszewski M.B., Ekblad E. (2005). Effects of vasoactive intestinal peptide and galanin on survival of cultured porcine myenteric neurons. Regul. Pept..

[20] Lang R., Gundlach A.L., Kofler B. (2007). The galanin peptide family: Receptor pharmacology, pleiotropic biological actions, and implications in health and disease. Pharmacol. Ther..

[21] Sarnelli G., Vanden Berghe P., Raeymaekers P., Janssens J., Tack J. (2004). Inhibitory effects of galanin on evoked [Ca^2+^] responses in cultured myenteric neurons. Am. J. Physiol. Gastrointest Liver Physiol..

[22] Sundler F., Ekblad E., Grunditz T., Håkanson R., Uddman R. (1988). Vasoactive intestinal peptide in the peripheral nervous system. Ann. N. Y. Acad. Sci..

[23] Ekblad E., Sundler F. (1997). Distinct receptors mediate pituitary adenylate cyclase-activating peptide- and vasoactive intestinal peptide-induced relaxation of rat ileal longitudinal muscle. Eur. J. Pharmacol..

[24] Sandgren K., Lin Z., Fex Svenningsen A., Ekblad E. (2003). Vasoactive intestinal peptide and nitric oxide promote survival of adult rat myenteric neurons in culture. J. Neurosci. Res..

[25] Roza C., Reeh P.W., Substance P. (2001). Calcitonin gene related peptide and PGE2 co-released from the mouse colon: A new model to study nociceptive and inflammatory responses in viscera, in vitro. Pain..

[26] Grider J.R. (1994). CGRP as a transmitter in the sensory pathway mediating peristaltic reflex. Am. J. Physiol..

[27] Maggi C.A., Giuliani S., Zagorodnyuk V. (1996). Calcitonin gene-related peptide (CGRP) in the circular muscle of guinea-pig colon: Role as inhibitory transmitter and mechanisms of relaxation. Regul. Pept..

[28] Holzer P., Barthó L., Matusák O., Bauer V. (1989). Calcitonin gene-related peptide action on intestinal circular muscle. Am. J. Physiol..

[29] Palus K., Całka J. (2016). Neurochemical Plasticity of the Coeliac-Superior Mesenteric Ganglion Complex Neurons Projecting to the Prepyloric Area of the Porcine Stomach following Hyperacidity. Neural. Plast..

[30] Callaghan B.C., Cheng H.T., Stables C.L., Smith A.L., Feldman E.L. (2012). Diabetic neuropathy: Clinical manifestations and current treatments. Lancet Neurol..

[31] Chandrasekharan B., Srinivasan S. (2007). Diabetes and the enteric nervous system. Neurogastroenterol. Motil..

[32] Yarandi S.S., Srinivasan S. (2014). Diabetic gastrointestinal motility disorders and the role of enteric nervous system: Current status and future directions. Neurogastroenterol. Motil..

[33] Al-Awar A., Kupai K., Veszelka M., Szűcs G., Attieh Z., Murlasits Z., Török S., Pósa A., Varga C. (2016). Experimental Diabetes Mellitus in Different Animal Models. J. Diabetes Res..

[34] Wolf E., Braun-Reichhart C., Streckel E., Renner S. (2014). Genetically engineered pig models for diabetes research. Transgenic Res..

[35] Bulc M., Palus K., Zielonka Ł., Gajęcka M., Całka J. (2017). Changes in expression of inhibitory substances in the intramural neurons of the stomach following streptozotocin- induced diabetes in the pig. World J. Gastroenterol..

[36] Bulc M., Palus K., Dąbrowski M., Całka J. (2019). Hyperglycaemia-Induced Downregulation in Expression of nNOS Intramural Neurons of the Small Intestine in the Pig. Int. J. Mol. Sci..

[37] Bulc M., Palus K., Całka J., Zielonka Ł. (2018). Changes in Immunoreactivity of Sensory Substances within the Enteric Nervous System of the Porcine Stomach during Experimentally Induced Diabetes. J. Diabetes Res..

[38] Larsen M.O., Rolin B. (2004). Use of the Goettingen Minipig as a Model of Diabetes with Special Focus on Type 1 Diabetes Research. ILAR..

[39] Larsen M.O., Wilken M., Gotfredsen C.F., Carr R.D., Svendsen O., Rolin B. (2002). Mild streptozotocin diabetes in the Gottingen minipig. A novel model of moderate insulin deficiency and diabetes. Am. J. Physiol. Endocrinol. Metab..

[40] Fricker J. (2001). The pig: A new model of diabetic atherosclerosis. Drug Discov. Today..

[41] Rosenfeld L. (2002). Insulin: Discovery and controversy. Clin. Chem..

[42] Alberti K.G., Zimmet P.Z. (1998). Definition, diagnosis and classification of diabetes mellitus and its complications. Part 1: Diagnosis and classification of diabetes mellitus provisional report of a WHO consultation. Diabet Med..

[43] Rodrigues M.L., Motta M.E. (2012). Mechanisms and factors associated with gastrointestinal symptoms in patients with diabetes mellitus. J. Pediatr (Rio J.).

[44] King A.J. (2012). The use of animal models in diabetes research. Br. J. Pharmacol..

[45] Schranz D.B., Lernmark A. (1998). Immunology in diabetes: An update. Diabetes Metab. Rev..

[46] Papatheodorou K., Papanas N., Banach M., Papazoglou D., Edmonds M. (2016). Complications of Diabetes 2016. J. Diabetes Res..

[47] Forbes J.M., Cooper M.E. (2013). Mechanisms of diabetic complications. Physiol. Rev..

[48] Xie Z., Chang C., Zhou Z. (2014). Molecular mechanisms in autoimmune type 1 diabetes: A critical review. Clin Rev. Allergy Immunol..

[49] Like A.A., Rossini A.A. (1976). Streptozotocin-induced pancreatic insulitis: New model of diabetes mellitus. Science.

[50] Palus K., Bulc M., Całka J. (2018). Changes in VIP-, SP- and CGRP- like immunoreactivity in intramural neurons within the pig stomach following supplementation with low and high doses of acrylamide. Neurotoxicology.

[51] Skobowiat C., Calka J., Majewski M. (2011). Axotomy induced changes in neuronal plasticity of sympathetic chain ganglia (SChG) neurons supplying descending colon in the pig. Exp. Mol. Pathol..

[52] Ballmann M., Conlon J.M. (1985). Changes in the somatostatin, substance P and vasoactive intestinal polypeptide content of the gastrointestinal tract following streptozotocin-induced diabetes in the rat. Diabetologia.

[53] Nowak T.V., Chey W.W., Chang T.M., Weisbruch J.P., Fouquet G. (1995). Effect of streptozotocin-induced diabetes mellitus on release of vasoactive intestinal polypeptide from rodent small intestine. Dig. Dis. Sci..

[54] Whittaker V.P. (1989). Vasoactive intestinal polypeptide (VIP) as a cholinergic co-transmitter: Some recent results. Cell Biol. Int. Rep..

[55] Nasef N.A., Mehta S., Ferguson L.R. (2017). Susceptibility to chronic inflammation: An update. Arch. Toxicol.

[56] Brenneman D.E., Philips T.M., Hauser J., Hill J.M., Spong C.Y., Gozes I. (2003). Complex array of cytokines released by vasoactive intestinal pep-tide. Neuropeptides.

[57] El-Salhy M. (1998). Neuroendocrine peptides of the gastrointestinal tract of an animal model of human type 2 diabetes mellitus. Acta Diabetol..

[58] El-Salhy M. (2001). Gastrointestinal transit in nonobese diabetic mouse: An animal model of human diabetes type 1. J. Diabetes Complicat..

[59] Zawada A.E., Moszak M., Skrzypczak D., Grzymisławski M. (2018). Gastrointestinal complications in patients with diabetes mellitus. Adv. Clin Exp. Med..

[60] Heinricher M.M. (2016). Pain Modulation and the Transition from Acute to Chronic Pain. Adv. Exp. Med. Biol..

[61] Belai A., Calcutt N.A., Carrington A.L., Diemel L.T., Tomlinson D.R., Burnstock G. (1996). Enteric neuropeptides in streptozotocin-diabetic rats; effects of insulin and aldose reductase inhibition. J. Auton Nerv. Syst..

[62] Belai A., Lincoln J., Burnstock G. (1987). Lack of release of vasoactive intestinal polypeptide and calcitonin gene-related peptide during electrical stimulation of enteric nerves in streptozotocindiabetic rats. Gastroenterology.

[63] Belai A., Burnstock G. (1990). Changes in adrenergic and peptidergic nerves in the submucous plexus of streptozocin-diabetic rat ileum. Gastroenterology.

[64] Marchand L., Kawasaki-Ogita Y., Place J., Fayolle C., Lauton D., Boulet F., Farret A., Renard E. (2017). Long-Term Effects of Continuous Subcutaneous Insulin Infusion on Glucose Control and Microvascular Complications in Patients With Type 1 Diabetes. J. Diabetes Sci. Technol..

[65] Qu Z.D., Thacker M., Castelucci P., Bagyánszki M., Epstein M.L., Furness J.B. (2008). Immunohistochemical analysis of neuron types in the mouse small intestine. Cell Tissue Res..

[66] Pidsudko Z., Wąsowicz K., Kaleczyc J., Majewski M., Lakomy M. (2008). Proliferative enteropathy induced changes in expression of DβH, VAChT and NOS in the neurons on intramural ganglia of the porcain ileum. Veterin Med..

[67] Monckton G., Pehowich E. (1980). Autonomic neuropathy in the streptozotocin diabetic rat. Can. J. Neurol. Sci..

[68] Spangeus A., Suhr O., El-Salhy M. (2000). Diabetic state affects the innervation of gut in an animal model of human type 1 diabetes. Histol. Histopathol..

[69] LePard K.J. (2005). Choline acetyltransferase and inducible nitric oxide synthase are increased in myenteric plexus of diabetic guinea pig. Auton. Neurosci..

